# Supplementary material for: Common and phylogenetically widespread coding for peptides by bacterial small RNAs
Source: BMC Genomics. 2017 Jul 21;18:553. doi: 10.1186/s12864-017-3932-y (PMC5521070; doi:10.1186/s12864-017-3932-y)
Supplement: Supplementary file 1 — Supplemental figures. (PDF 1870 kb) [file 12864_2017_3932_MOESM1_ESM.pdf]

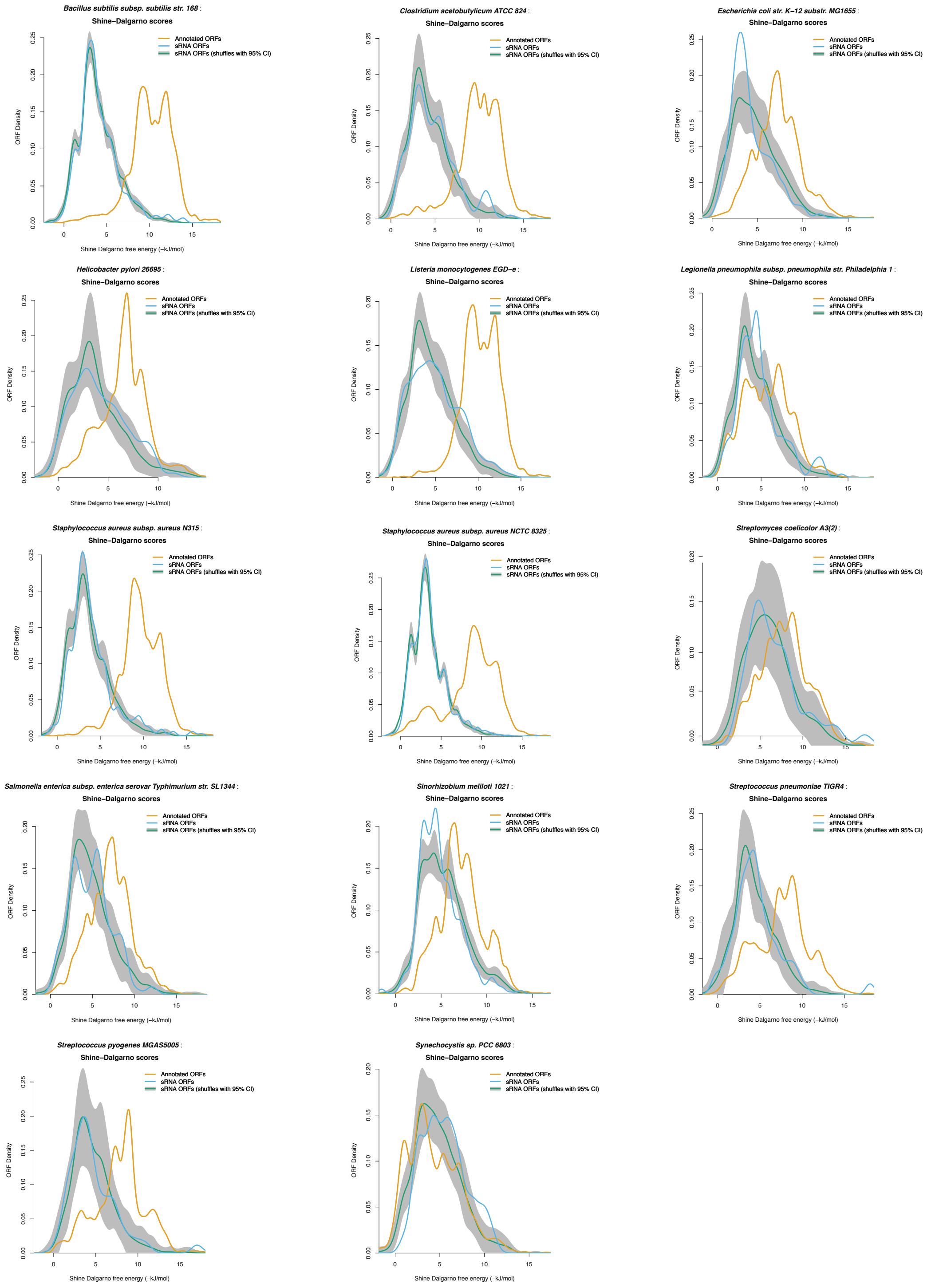

Supplementary figure 1: SD score distributions for individual bacterial strains. Each histogram compares the Shine-Dalgarno scores (representing the strength of binding to the ribosome) of full-length annotated ORFs, sRNA ORFs, and mock sRNA ORF controls. Higher scores (in -kJ/mol) indicate stronger ribosomal recruitment. The grey band around the mock ORFs represent 95% confidence intervals, so deviations of the sRNA ORFs from this band represent nominally significant differences.

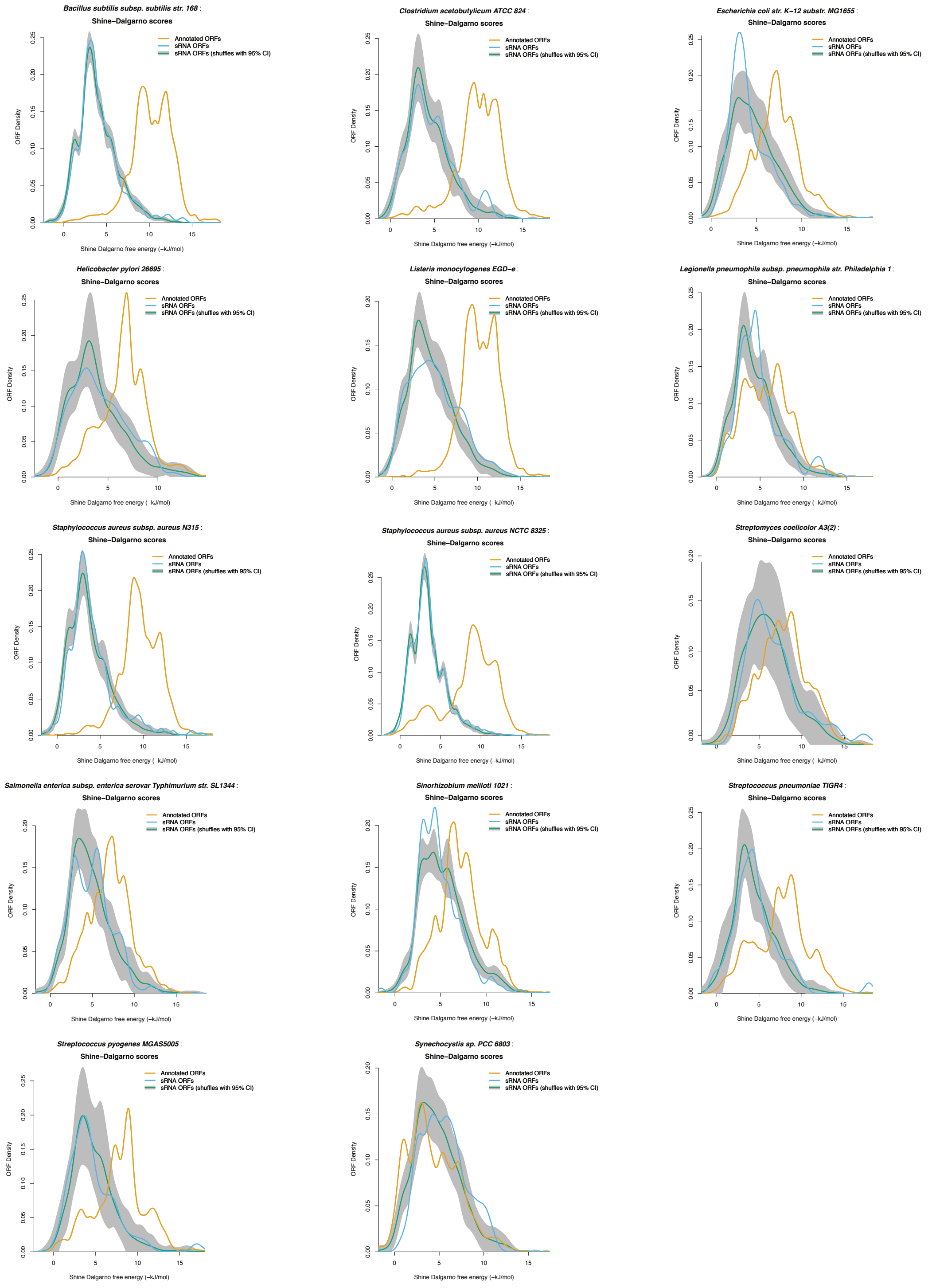

Supplementary figure 2: D_n/D_s score distributions for indivudual bacterial strains. Each cumulative density plot compares D_n/D_s likelihood scores (representing evidence for natural selection at the amino acid level) of full-length annotated ORFs, sRNA ORFs, and mock sRNA ORF controls. When some sRNA ORFs have significant evidence for natural selection at the amino acid level, the sRNA ORF curve is closer to the annotated ORF curve than the mock sRNA ORF curve is.

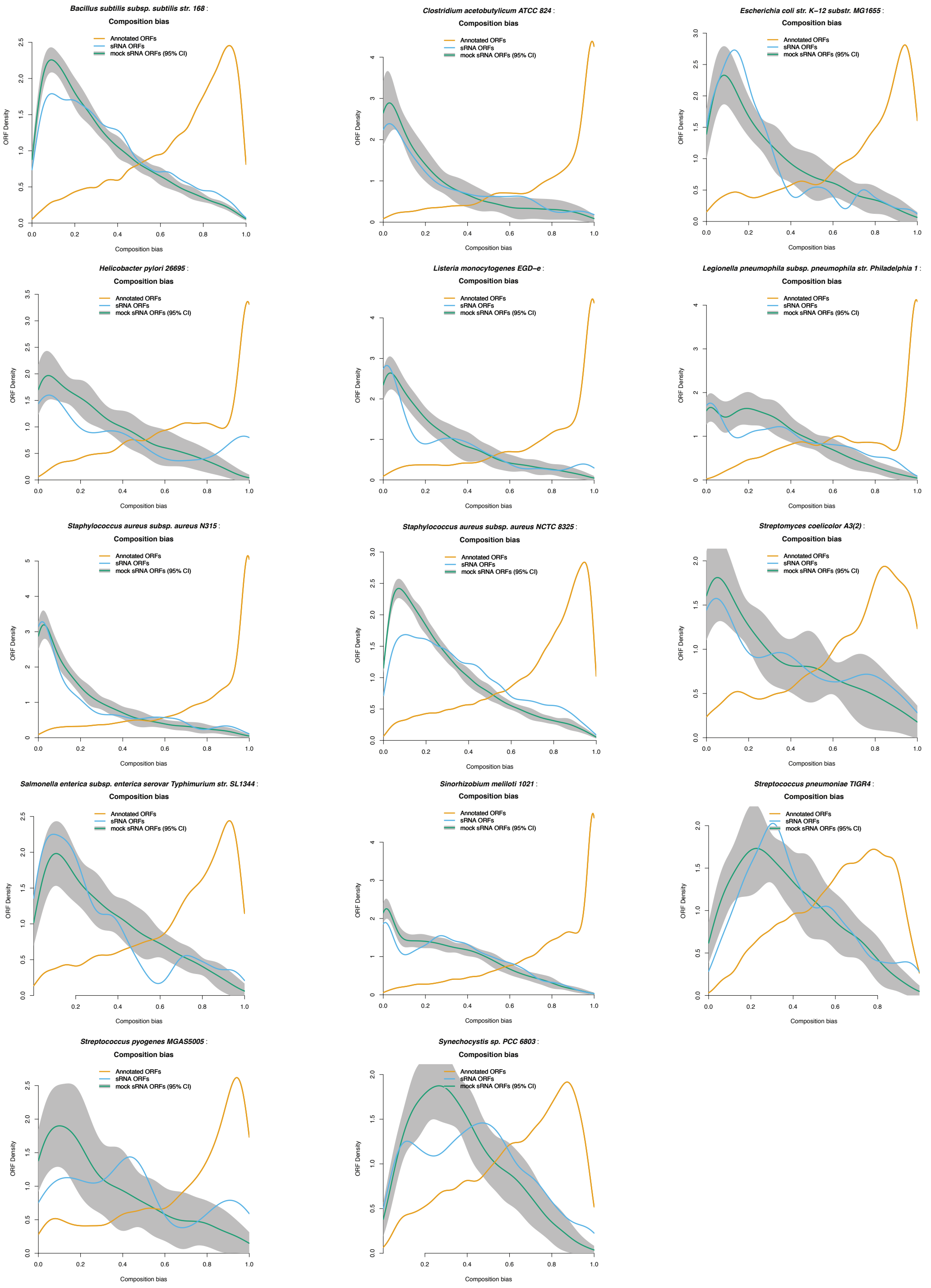

Supplementary figure 3: Composition bias score distributions for individual bacterial strains. Each histogram compares the nucleotide composition scores (representing phase-specific nucleotide evidence towards protein-coding) of full-length annotated ORFs, sRNA ORFs, and mock sRNA ORF controls. Higher scores indicate nucleotide, dinucleotide, and trinucleotide composition and phase-specificity that is more like protein-coding genes than like controls. The grey band around the mock ORFs represent 95% confidence intervals, so deviations of the sRNA ORFs from this band represent nominally significant differences.

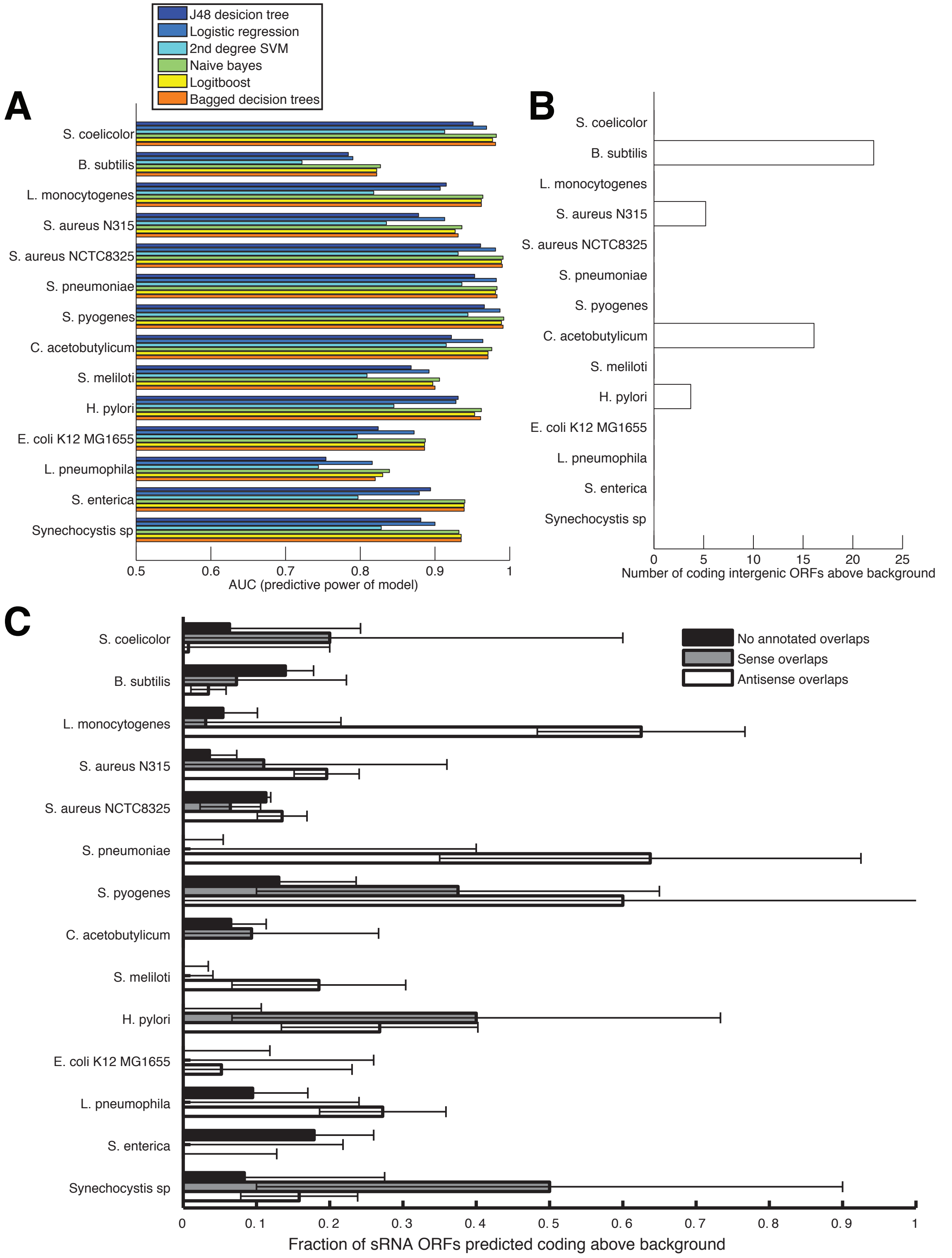

Supplementary figure 4: **A)** Accuracy of machine-learning classifiers as measured by AUC (area under the ROC curve) on 10-fold cross-validated training data. **B)** Number of intergenic ORFs predicted as coding above background without regard to sRNA annotations. **C)** Fraction of sRNA ORFs predicted

as coding above background for each species, broken down based on their overlap with annotated ORFs.

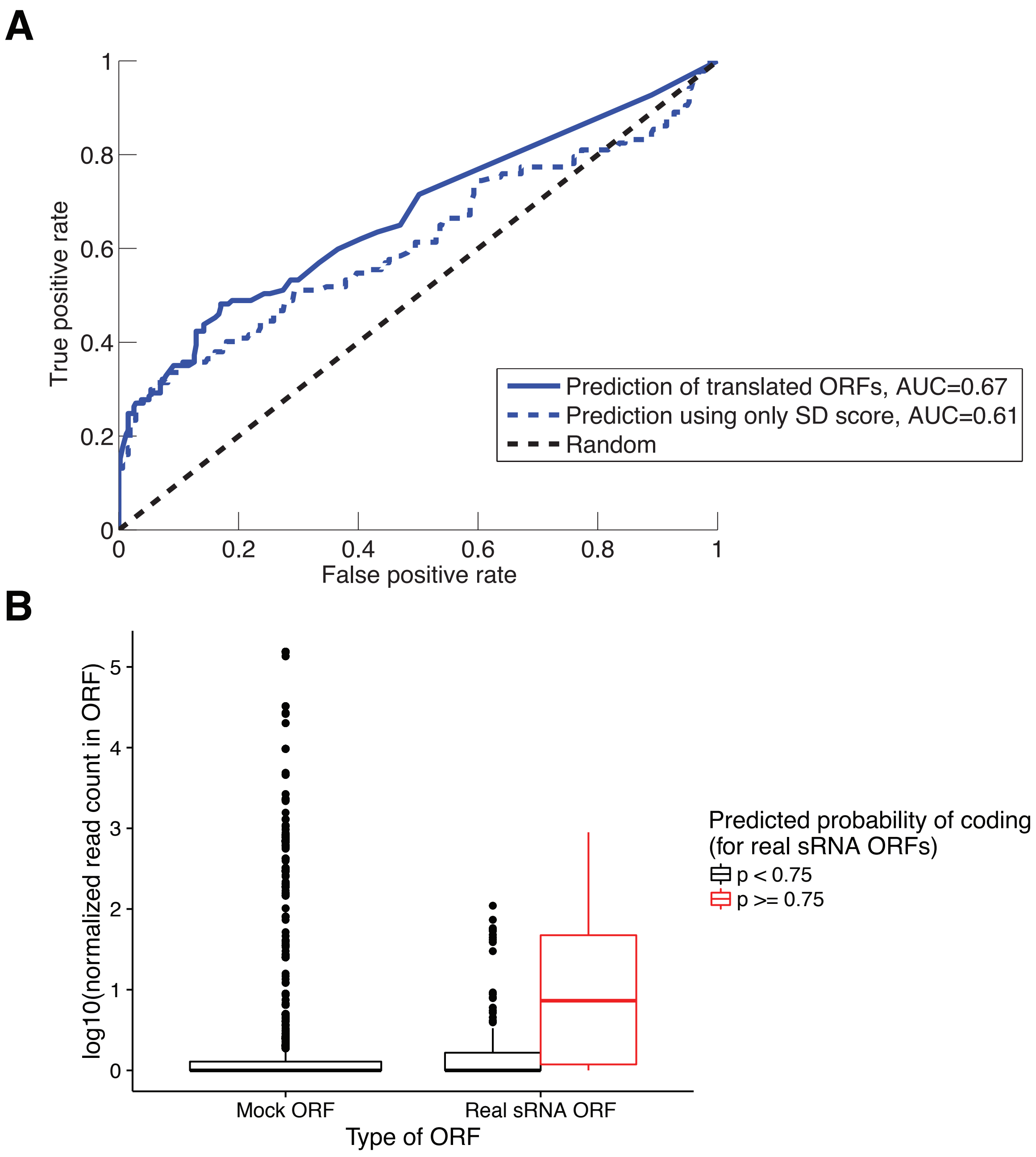

Supplementary figure 5: **A)** ROC curve for *B. subtilis* translated ORFs. sRNA ORFs with evidence for translation in ribosome profiling data were compared to mock ORFs using the coding score. Translated ORFs were predicted slightly better when using the SD score, the D_n/D_s score, and the nucleotide composition bias (solid blue line) than when using the SD score alone (broken blue line), as quantified by the area under the curve (AUC). **B)** Box plot showing log10 of read count (normalized by Li et al. 2012) summed over all positions in ORF for *B. subtilis* sRNAs and mock

sRNAs lacking overlap with annotated ORFs. Mock ORFs were chosen to match length and other sequence properties. Real sRNA ORFs were divided into those with coding score greater than 0.75 (n = 31, red) and those below (n = 134, black).

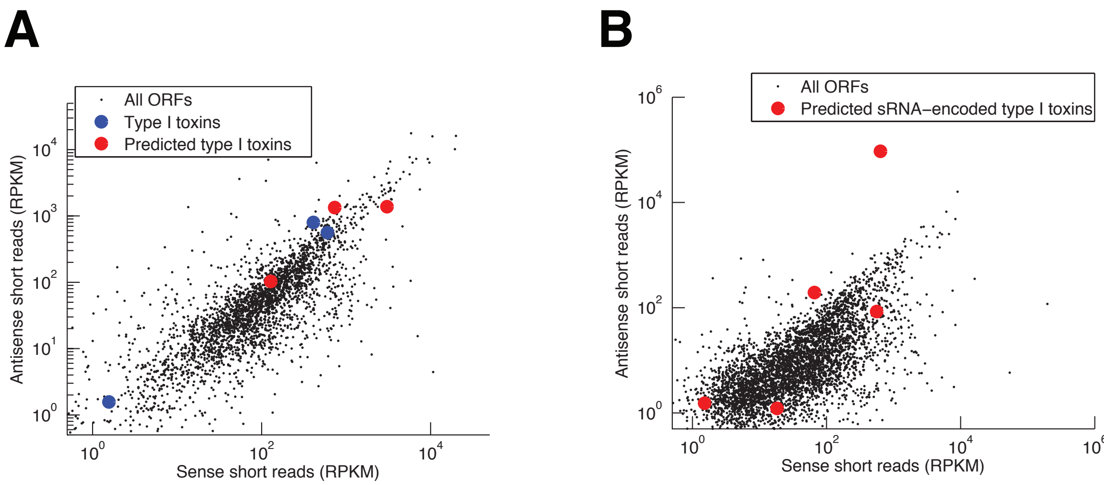

Supplementary figure 6: Short RNA degradation products. **A)** Short RNA reads mapping to the sense or antisense strand of *S. aureus* NCTC 8325 annotated ORFs. Type I toxins come from the BLAST annotations of Fozo et al., while the predicted type I toxins are intergenic ORFs with a coding score and type I toxin score of at least 0.5. **B)** As in **A)** but for *B. subtilis* 168 sRNA-encoded ORFs.
